# Supplementary material for: Irrigation and inequalities in the Senegal River Delta
Source: PLoS One. 2026 Apr 21;21(4):e0325862. doi: 10.1371/journal.pone.0325862 (PMC13098955; doi:10.1371/journal.pone.0325862)
Supplement: Attachment — Submitted filename: Response to reviewers.docx [file pone.0325862.s004.docx]

PLOS ONE Answers

| Number | Comments | Answers |
| --- | --- | --- |
| 1 | 1. Please ensure that your manuscript meets PLOS ONE's style requirements, including those for file naming. The PLOS ONE style templates can be found at  <https://journals.plos.org/plosone/s/file?id=wjVg/PLOSOne_formatting_sample_main_body.pdf> and  [https://journals.plos.org/plosone/s/file?id=ba62/PLOSOne_formatting_sample_title_authors_affiliations.pdf](https://track.editorialmanager.com/CL0/https:%2F%2Fjournals.plos.org%2Fplosone%2Fs%2Ffile%3Fid=ba62%2FPLOSOne_formatting_sample_title_authors_affiliations.pdf/1/010f0199a6371b12-e44df090-404a-48a8-9cd2-87c1613f87a2-000000/oG9yl3eeJtNdYMhty7kVmjTK_9volt4VoUS_80gVg50=231). | We have adapted the document to the journal's standards. The font sizes of the titles and subtitles have been changed, and the figures have been removed from the text and placed in a separate file. The scientific references follow the journal's citation and bibliographic style. |
| 2 | 2. Please provide details regarding participant consent. In the ethics statement in the Methods and online submission information, please ensure that you have specified (1) whether consent was informed and (2) what type you obtained (for instance, written or verbal, and if verbal, how it was documented and witnessed). If your study included minors, state whether you obtained consent from parents or guardians. If the need for consent was waived by the ethics committee, please include this information. | We obtained verbal consent from all our respondents before the start of the survey. They were also informed that participation in the survey was voluntary and that they could decide to stop the interview at any time. No minors were surveyed. |
| 3 | 3. We note that your Data Availability Statement is currently as follows: [All relevant data are within the manuscript and its Supporting Information files.]  Please confirm at this time whether or not your submission contains all raw data required to replicate the results of your study. Authors must share the “minimal data set” for their submission. PLOS defines the minimal data set to consist of the data required to replicate all study findings reported in the article, as well as related metadata and methods (<https://journals.plos.org/plosone/s/data-availability#loc-minimal-data-set-definition>). | We have modified the sentence, it should be noted that: Data used in this study are available on a reasonable request to the corresponding author |
| 4 | 4. We note that Figure 1 in your submission contain [map/satellite] images which may be copyrighted. All PLOS content is published under the Creative Commons Attribution License (CC BY 4.0), which means that the manuscript, images, and Supporting Information files will be freely available online, and any third party is permitted to access, download, copy, distribute, and use these materials in any way, even commercially, with proper attribution. For these reasons, we cannot publish previously copyrighted maps or satellite images created using proprietary data, such as Google software (Google Maps, Street View, and Earth). For more information, see our copyright guidelines: <http://journals.plos.org/plosone/s/licenses-and-copyright>. | Figure 1 is based on the authors' own data. We grant permission to PLOS ONE, its readers, and anyone interested to use it, and most importantly, to cite this work when it is published. |
| 5 | Please remove your figures from within your manuscript file, leaving only the individual TIFF/EPS image files, uploaded separately. These will be automatically included in the reviewers’ PDF | All figures have been removed from the text and uploaded as a separate file |
| 6 | If the reviewer comments include a recommendation to cite specific previously published works, please review and evaluate these publications to determine whether they are relevant and should be cited. There is no requirement to cite these works unless the editor has indicated otherwise. | The reviewer did not recommend citing previously published work. However, we have improved the context and discussion by adding new scientific references. |
| 7 | This manuscript addresses a relevant and timely topic: inequalities in land access and agricultural income among irrigated farmers in the Senegal River Delta. The data collected are valuable, the use of Gini and Lorenz analyses is appropriate, and the focus on an under-researched context adds originality to the work. | Thank you very much for this appreciation. We strongly believe that this study is very important in understanding the links between irrigated development and its role in social inequalities. Its publication will immerse the Senegal River Delta in a scientific debate that many countries have been engaged in for several years. |
| 8 | To fully meet the standards of a scientific publication in PLOS ONE, the manuscript would benefit from major revisions. While the study is well documented, its structure and tone currently resemble a technical report more than a scholarly article. Enhancing the clarity of the writing, deepening the analytical engagement, and strengthening the contextual framing — particularly with regard to institutional and policy dimensions — would significantly improve the paper. The following comments are offered with the intention of constructively supporting the authors in enhancing the overall quality and impact of their work. | Thank you very much for this pertinent comment, which enabled us to revise the entire article. We have made the necessary corrections, and the article now better conforms to the guidelines for a scientific paper rather than a technical report. |
| 9 | *The manuscript requires careful proofreading by a native English speaker or language editor. Issues such as incomplete sentences (e.g.,Discussion p. 9), repeated words (e.g., “UsuallyUsually”, p. 4), grammatical error (e.g., disapointed/disappointed) and unclear transitions affect readability and precision. | The English in the article was improved by Patrick Van Damme, one of the co-authors. He teaches courses in English at several European universities and has published over 100 articles in English. |
| 10 | *The text briefly mentions Senegal’s goal of food sovereignty by 2029 but does not elaborate on current national policies or programs related to rice production. A more developed discussion of policy efforts and institutional strategies would help situate the findings within present-day challenges and debates. | We have added a more in-depth analysis of the political efforts and institutional strategies that place these findings in the context of current challenges and debates. We have added this paragraph on page 10 as follows: This finding is widespread across Senegal and partly explains the government’s decision to develop the National Strategy for Food Sovereignty (SNSA) for the 2024–2028 period, which was adopted in January 2023. Among the corrective measures implemented by the State, two stand out as transformative in reshaping the country’s agricultural landscape and improving people’s access to staple foods. The first involves the introduction of rainfed rice cultivation into production systems and areas that had previously been excluded from rice farming. The second consists of introducing wheat production in Senegal, with the dual objective of reducing the country’s trade deficit and wheat imports, and ensuring better access and availability of wheat in local markets. The government’s ambitions under this new policy framework are particularly significant, aiming by 2025 to achieve full domestic coverage of rice needs and 42% coverage in locally produced wheat, alongside self-sufficiency in other key crops such as onions, potatoes, and carrots [35]. Such efforts would increase farmers' incomes, reduce inequalities among producers, and contribute to Senegal’s goal of achieving food sovereignty. |
| 11 | *SAED is mentioned but not defined or contextualized. Clarifying its status (e.g., government agency) and role in irrigation policy would be useful for non-specialist readers. Additionally, the ISRA — a major actor in agricultural research in Senegal — is not mentioned at all. If relevant, its role should be acknowledged to provide a more complete picture of the institutional landscape. | We have now outlined the role of SAED in the organization of irrigated rice cultivation. In northern Senegal, SAED is more involved and better equipped than ISRA. ISRA is more active in other regions of Senegal, which is why we believe it is not relevant to mention it in this study. Additional information on SAED is described as follows: "Rice cultivation in the hot off-season is usually carried out on collective hydro-agricultural plots, set up by SAED with adequate infrastructure. SAED is the public institution historically responsible for the development and organization of irrigation in the Senegal River valley and delta. SAED established a large number of collective perimeters equipped with adequate irrigation and drainage infrastructure, where most of the hot off-season rice cultivation (HOS) takes place. Each participating household has at least one plot and is well organized. |
| 12 | *The paper presents a useful snapshot of inequalities but would benefit from a more dynamic and process-oriented interpretation. For example, exploring how inequalities have emerged or been reproduced over time (e.g., through inheritance, reforms, market access) could deepen the analysis, even with cross-sectional data. | The discussion on inequality has been improved. We cited an article and showed how inequality in the Delta is structured and evolves over time.  Elouaamari and Lagarde (2025) emphasize that irrigation development in the Senegal River Delta has reproduced and, in some cases, deepened existing social and territorial inequalities. The authors show that access to irrigated land and water is highly uneven, shaped by historical policies, socio-economic status of farmers, and institutional arrangements. Large-scale irrigation schemes, often managed by public or private entities, tend to favour wealthier or better-connected farmers who can afford inputs, equipment, and administrative fees. In contrast, smallholders and young farmers are often relegated to marginal lands or excluded from formal irrigation networks [22]. (P7-8)  “ This institutional and technical asymmetry of the organization of irrigation in the delta is also described by Elouaamari and Lagarde (2025): the design of hydraulic infrastructures and water allocation systems prioritizes rice cultivation and production goals aligned with national food policies, rather than local livelihood diversification. This “top-down” production model has created dependency on centralized management structures, limiting farmers’ autonomy and adaptive capacity. Moreover, inequalities are reinforced by differences in access to crop advice, credit, and market opportunities, which contribute to the persistence of a dual agricultural system, one modern and capital-intensive, and the other traditional and precarious [22]. (P9) |
| 13 | *The analysis remains largely descriptive. To strengthen the paper’s academic contribution, the authors could more explicitly connect their findings to broader theoretical or structural debates on inequality, rural development, and irrigation governance. | We have made the entire paper less descriptive and more scientific. We have added six new scientific references and improved the quality of the discussion. This allows us to better highlight the inequalities that structure irrigated agriculture. |
| 14 | *While the quantitative data are valuable, the narrative does not always reference or explain tables and figures clearly (e.g., tables 2 and 3). Improving the integration of visual elements into the text would enhance clarity. | We have integrated the images and tables into the table by announcing and interpreting them. The journal asked us to remove the figures from within the text and put them in a separate file. However, they are still referenced in the parts of the text where they should be inserted. |
| 15 | *The study treats households as a homogeneous unit, without exploring variables such as gender, age, or ethnicity, which are often relevant to understanding inequality. While this may be beyond the current scope, acknowledging it as a limitation would strengthen the manuscript. | Yes, it would be very relevant to add these variables for the analysis of inequalities, especially gender and ethnicity. We will take this into account in our future publications and mention it as a limitation of this study.  “This study examined inequality at the household level; however, other dimensions of inequality might emerge if variables such as gender, age, or ethnicity of producers were considered. This represents a limitation of the current analysis and an opportunity for future research to explore these additional factors » P 10 |
| 16 | *In line with PLOS ONE’s open science standards, it would be helpful to indicate whether the dataset can be shared publicly (even in anonymized form), or to explain any constraints to doing so. | We have added it to the Plos One platform. The data are available upon request to the lead author. |
| 17 | I commend the authors for their fieldwork in a challenging context and for addressing a subject of critical importance. With substantial revisions to enhance the manuscript’s clarity, analytical depth, and contextual grounding, this study has the potential to make a valuable contribution to the literature on irrigation and inequality in West Africa. I hope these comments are helpful and supportive of the authors’ continued work on this topic. | We would like to thank the reviewer for the quality of their contributions, their insightful comments, and above all, the new dimension that our article has taken on after incorporating their suggestions. We also thank them for their availability and the valuable time they devoted to reviewing this manuscript. |
