# Supplementary material for: Irrigation and inequalities in the Senegal River Delta
Source: PLoS One. 2026 Apr 21;21(4):e0325862. doi: 10.1371/journal.pone.0325862 (PMC13098955; doi:10.1371/journal.pone.0325862)
Supplement: Attachment — Submitted filename: 1 answers to reviewers.docx [file pone.0325862.s005.docx]

Revision article irrigation et inégalités dans le delta du fleuve Sénégal

| Reviseurs | Commentaires | Réponses |
| --- | --- | --- |
| Réviseur 1 | Thank you for the careful and constructive revision of the manuscript. The authors have addressed all reviewer comments in a substantive way, leading to a clearer, more analytically grounded, and better contextualized paper. I consider the manuscript ready for publication | We sincerely thank the reviewer for the positive assessment and for acknowledging the improvements made to the manuscript. We are pleased that the paper is now considered ready for publication. |
| Réviseur 2 | Thank you for submitting this important research on irrigation inequalities in the Senegal River Delta. The study addresses a critical issue affecting agricultural development in West Africa, and your fieldwork with 326 households provides valuable empirical evidence. The use of Gini coefficients and Lorenz curves is appropriate for analyzing income distribution, and the finding that 72% of households cultivate less than 5 hectares while facing significant income deficits highlights serious equity concerns. | We thank the reviewer for these encouraging remarks and for recognizing the relevance of our methodological approach. We agree that the concentration of land and income deficits among the 72% of smallholders (cultivating <5 ha) is the most critical finding of this study. It underscores the structural barriers that prevent small-scale farmers from achieving economic viability in the Delta. By quantifying these inequalities using Gini coefficients and Lorenz curves, we aimed to provide a clear baseline for policymakers to address these disparities through more inclusive agricultural strategies |
|  | The revisions have improved the manuscript considerably, particularly the expanded discussion of SAED's role and the addition of recent policy context. | We are grateful to the reviewer for acknowledging the improvements made to the discussion of the institutional and policy framework. By further detailing the role of SAED and aligning our findings with recent national agricultural strategies, we aimed to provide a more comprehensive understanding of how institutional management directly influences socio-economic disparities. We believe this addition strengthens the practical implications of our study for land governance and irrigation management in the region. |
|  | Comment 1: In the Methods section (pages 10-11, around line 174), you mention that villages were selected based on geographical position, ethnic group, and demographic weight. Could you explain more clearly how you actually chose the 20 specific villages from all possible villages in Diama and Ross Béthio? Did you use a systematic approach, or were there practical considerations that guided this selection? This would help readers understand if your sample might be biased in any particular direction. | We have clarified the sampling process in the "Methods" section. The 20 villages were selected using a stratified purposeful sampling approach. From the comprehensive list of settlements in the Diama and Ross Béthio districts provided by the SAED local delegations and the municipalities, we stratified villages based on their demographic size and ethnic composition (to ensure representation of Wolof, Peulh, and Moor communities). Within each stratum, villages were selected to cover the geographic diversity of the Delta (upstream vs. downstream zones). |
|  | Comment 2: On page 11 where you describe the income calculation formula (equation 1), I'm wondering if you could discuss what expenses were included? For example, did you count labor costs, land rental, water fees, or only material inputs like seeds and fertilizers? Also, how did you handle households that use family labor versus hired labor - were these treated differently in the expense calculations? | We thank the reviewer for this insightful comment on the rigor of our income calculations. In Equation 1, the agricultural income was calculated as the gross value of production minus all explicit operating costs. Specifically, the expenses included:  Material inputs: Seeds, chemical fertilizers (urea and DAP), and herbicides.  Service costs: Mechanical plowing, harvesting (combine harvester), and water fees paid to the SAED or local water unions (GIE).  Labor costs: We made a clear distinction between hired and family labor. Hired labor (seasonal workers for transplanting or weeding) was included as a direct cash expense. Family labor, however, was not monetized or deducted from the net income in this specific study, following a common practice in smallholder farming systems where family labor is considered an internal resource rather than an accounting cost.  Land rental: Only actual cash payments for land leasing were recorded for households that did not own their land rights.  We have added a clarifying paragraph in the "Methods" section (Equation 1 description) to explicitly list these inclusions/exclusions, ensuring the reader can better interpret the Gini coefficients based on these net income values. |
|  | Comment 3: Looking at Table 1 (page 11), you show that 7% of households farm 20 hectares or more. In the text you mention that some of these have over 100 hectares. Can you provide more detail about this group - roughly how many households fall into this very large category, and are they primarily private companies, cooperatives, or wealthy individual farmers? Understanding who these large operators are would help contextualize the inequality patterns. | We thank the reviewer for this request to further contextualize the "large operator" category. In our sample of 326 households, approximately 7% cultivate 20 hectares or more. Within this group, a distinct elite category emerges: households cultivating over 100 hectares, which represent 1% of our total sample.  These very large-scale operators are not primarily private companies or cooperatives, but rather wealthy individual investors. Based on our field qualitative insights, this group is composed of:  (i)High-ranking government officials and civil servants, (ii) Religious dignitaries (Marabouts) who hold significant land and social influence, (ii) Urban-based businessmen, physicians, and private investors from Dakar and Saint-Louis.  These individuals leverage their financial capital and social networks to invest in irrigation, contrasting sharply with the 72% of smallholders who lack such resources. We have updated the "Results" and "Discussion" sections to include these details, as they are crucial for understanding the "urban-investor" phenomenon that drives land concentration in the Senegal River Delta. |
|  | Comment 4: The three growing seasons are described on page 12 (around lines 233-238), but the date ranges seem to overlap. For instance, you say hot off-season is February-June and also March-June in different places. Could you provide a clearer table or figure showing the exact months for each season and how they connect? This would help readers unfamiliar with the region understand the agricultural calendar better. | We apologize for the overlapping dates in the previous version, which may have caused confusion. We have now standardized the agricultural calendar throughout the manuscript to reflect the official cycles in the Senegal River Delta as defined by SAED.  To ensure clarity for readers unfamiliar with the region, we have added a new Table 1: Agricultural Calendar in the Senegal River Delta, which clearly defines the three seasons:   1. Hot Dry Off-Season (Contre-saison chaude): March to June. 2. Rainy Season (Hivernage): July to October/November. 3. Cold Dry Off-Season (Contre-saison froide): November to February (primarily dedicated to market gardening or a second cereal cycle in some schemes).   We have also synchronized all text references (notably on pages 12 and 15) to match these ranges, ensuring a consistent narrative regarding when households invest in inputs and harvest their crops. |
|  | Comment 5: In Table 2 (page 12), you show that 67.75% of cultivated area is in hot off-season, but then you mention that 32% of land is not exploited even during this peak season (page 13, discussion section). Could you clarify this apparent contradiction? Are you referring to different land categories, or is there a calculation I'm misunderstanding? | As specified on page 11 of the manuscript, the 32% of land that remains unplanted during the peak hot off-season is not a statistical discrepancy but a core finding of our study.  This "land dormancy" (32%) is driven by a combination of two factors:  Financial barriers: Smallholders often lack the liquid capital to cover high input and water costs for their entire landholding.  Environmental degradation: As noted in our text, soil salinization and falling yields lead to the partial or total abandonment of certain plots.  Therefore, while the hot off-season represents the peak of annual activity (67.75% of the total annual cropped area), nearly one-third of the potential irrigable land remains unproductive. This highlights a "double penalty" for smallholders: they possess limited land, and a significant portion of that land is becoming unusable or unaffordable to cultivate due to environmental and financial stress. We have ensured this distinction is now even more explicit in the revised discussion. |
|  | Comment 6: Your Gini coefficient results in Table 4 (page 14) are quite high, ranging from 0.85 to 0.94. How do these values compare to other agricultural regions in Senegal or similar irrigation schemes in West Africa? Providing some comparative context would help readers assess whether the Delta situation is exceptionally unequal or reflects broader regional patterns. | The Gini coefficients observed in this study (0.85 for land and 0.94 for income) indicate a level of inequality significantly higher than the average for sub-Saharan African agriculture, where land Ginis typically hover between 0.45 and 0.65. This extreme disparity is characteristic of the Senegal River Delta’s specific political economy, where irrigation serves as a vehicle for wealth concentration rather than just poverty reduction. We have included in the discussion a study on the Office du Niger that explains the same phenomenon. It should also be noted that studies using the Gini index in West Africa are rare |
|  | Comment 7: In the discussion section around page 15, you cite several studies from India, Nigeria, and Brazil to support your findings. While these are relevant, I noticed fewer references to other irrigation schemes in francophone West Africa. Are there studies from Mali's Office du Niger or Mauritania's irrigation areas that might provide more direct comparisons? If such studies don't exist, mentioning this gap could strengthen your contribution. | We fully agree with the reviewer that regional comparisons within Francophone West Africa provide a more direct and culturally relevant context than those from India or Brazil. We have significantly revised the Discussion (page 15) to incorporate findings from the Office du Niger in Mali |
|  | Comment 8: Figure 5 presents trends from 2001-2021, showing declining rice yields from 7.28 T/ha to 5.55 T/ha. This is a dramatic drop, but the text doesn't really explain why yields are falling so sharply. Could you add some discussion about the possible causes - is it soil degradation, reduced fertilizer use, water management problems, or other factors? This seems like a critical issue that deserves more attention. | The decline in average yields from 7.28 T/ha in 2001 to 5.55 T/ha in 2021 is indeed a critical issue that reflects the "sustainability crisis" of the intensification model in the Delta. We have added a dedicated paragraph in the Discussion to explain this drop, attributing it to four main factors: |
|  | Comment 9: On page 16 in the conclusion, you recommend that the government should "impose a results contract on large producers and define a minimum crop intensity to be achieved by all operators." Could you elaborate on what you mean by "results contract" and how this would work practically? | We thank the reviewer for this opportunity to clarify our policy recommendations. By "results contract" (or performance covenant), we propose a regulatory shift from land ownership to land productivity. Practically, this would involve: Performance-Based Land Tenure and Water-Access Linking. We add a sentence in the conclusion |
|  | Comment 10: Regarding your data availability statement, you now say data are available "on reasonable request" to the corresponding author. Given that your data appear to be fully anonymized (no personally identifiable information), is there a specific reason why you cannot deposit the dataset in a public repository like Figshare or Zenodo? Making the data more openly available would increase the transparency and potential impact of your work. | We fully agree that data sharing increases the impact and transparency of research. While we initially indicated availability "on reasonable request," we have complied with the journal's requirements. The fully anonymized dataset supporting the findings of this study has been submitted directly to the PLOS ONE editorial office.  Regarding public repositories like Figshare or Zenodo, we have ensured that all the underlying data required to replicate the Gini and Lorenz calculations are included in the Supporting Information files (S1 Data) accompanying this manuscript. This ensures long-term accessibility and transparency while respecting our institutional data management protocols. |
| Reviewer 3 | title doesn't match with the included subject Imported missing data 1-weather condition to be gathered in one table for different seasons | We have added a summary of the Sahelian climate cycles (Dry/Hot, Rainy, Dry/Cold) with temperature and evapotranspiration averages. |
|  | 2- type of irrigation system | We have clarified that the system is based on large-scale hydraulic infrastructure managed by SAED, utilizing electric and diesel pumping stations to divert water from the Senegal River into primary and secondary canals. |
|  | 3-Type of soil under study physical or chemical soil properties | The soils are predominantly hydromorphic vertisols with high clay content (>40%), characterized by high water retention but high tillage resistance. |
|  | 4-howmuch water needed for rice cultivation under irrigation | Following SAED standards, rice water needs in the Delta are estimated between 10,000 and 12,000 m³/ha per season, depending on the period. |
|  | 5- Figures are not clear | We have adhered to the standards for scientific publications regarding image quality. We hope that the journal PLOS ONE will be able to guide us or help us improve the quality of our images. |
|  | 6- In figure 2 you write cultivated and compare with those uncultivated . | Yes, the graph shows, for each season, those who farmed and those who did not farm in our sample. That is why, in our discussion, we highlighted the uncultivated land and the farmers who do not go out to work in the fields as a shortfall. The caption for Figure 2 was misplaced; we have moved it to the correct location. |
